# Health-related quality of life of COVID-19 two and 12 months after intensive care unit admission

**DOI:** 10.1186/s13613-022-00991-0

**Published:** 2022-02-20

**Authors:** Alexandre Demoule, Elise Morawiec, Maxens Decavele, Raphaelle Ohayon, Roxane Malrin, Maria Alejandra Galarza-Jimenez, Pierantonio Laveneziana, Capucine Morelot-Panzini, Thomas Similowski, Yann De Rycke, Jesus Gonzalez-Bermejo

**Affiliations:** 1 AP-HP, Groupe Hospitalier Universitaire APHP-Sorbonne Université, Site Pitié-Salpêtrière, Service de Médecine Intensive Et Réanimation (Département R3S), 75013 Paris, France; 2 Sorbonne Université, INSERM, UMRS1158, Neurophysiologie Respiratoire Expérimentale Et Clinique, 75005 Paris, France; 3 Sorbonne Université, GRC 30, REanimation Et Soins Intensifs du Patient en Insuffisance Respiratoire aigüE, AP-HP, Hôpital de La Pitié-Salpêtrière, 75013 Paris, France; 4 AP-HP, Groupe Hospitalier Universitaire APHP-Sorbonne Université, Site Pitié-Salpêtrière, Soins de Suite Et Réadaptation Respiratoires Et Neurorespiratoires (Département R3S), 75013 Paris, France; 5 AP-HP, Groupe Hospitalier Universitaire APHP-Sorbonne Université, Site Pitié-Salpêtrière, Service de Pneumologie (Département R3S), 75013 Paris, France; 6 AP-HP, Groupe Hospitalier Universitaire APHP-Sorbonne Université, Site Pitié-Salpêtrière, Service des Explorations Fonctionnelles de La Respiration, de L’exercice et de La Dyspnée (Département R3S), 75013 Paris, France; 7 AP-HP, Groupe Hospitalier Universitaire APHP-Sorbonne Université, Site Pitié-Salpêtrière, Département R3S, 75013 Paris, France; 8 Département Biostatistique Santé Publique Et Information Médicale, Centre de Pharmacoépidémiologie (Cephepi), Groupe Hospitalier Universitaire APHP-Sorbonne Université, Site Pitié-Salpêtrière, Paris, France; 9 Sorbonne Université, INSERM, Institut Pierre Louis d’Epidémiologie Et de Santé Publique, CIC-1421, 75013 Paris, France; 10 Service de Médecine Intensive–Réanimation, Hôpital Pitié-Salpêtrière, 47-83 Boulevard de l’Hôpital, 75651 Paris Cedex 13, France

**Keywords:** Pulmonary function tests, Dyspnea, Health-related quality of life, Mechanical ventilation, Mortality, Six-minute walk test, Exercise capacity, Acute respiratory distress syndrome, COVID-19

## Abstract

**Purpose:**

To describe health-related quality of life (HRQoL) and dyspnea of COVID-19, 2 and 12 months after an intensive care unit (ICU) stay**.**

**Methods:**

Patients discharged from the ICU between April and June 2020 and subsequently transferred to an inpatient rehabilitation facility were assessed 2 months and 12 months after ICU admission. HRQoL was assessed by the EuroQoL EQ-5D-3L (visual analog scale and time trade-off normalized to the French population algorithm) and dyspnea was assessed by the modified Medical Research Council (mMRC) dyspnea scale.

**Results:**

We enrolled 94 patients. Median EQ-5D-3L time trade-off was 0.80 (interquartile range, 0.36–0.91) at 2 months and 0.91 (0.52–1.00) at 12 months (*P* = 0.12). EQ-5D-3L visual analog scale was 70 (60–85) at 2 months and 70 (60–85) at 12 months (*P* = 0.07). The mMRC dyspnea scale was 3 (2–4) at ICU discharge, 1 (0–2), *P* < 0.001 at 2 months and 1 (1–2) at 12 months. At 12 months, 68 (76%) patients reported at least one symptom that was not present prior to ICU admission and 27 (61%) of the 44 patients who were previously working had returned to work. On multiple linear regression, factors associated with EQ-5D-3L were body mass index on ICU admission, tracheostomy, male gender and active smoking.

**Conclusions:**

Twelve months after ICU admission for COVID-19 and subsequent rehabilitation, a substantial proportion of patients reported alterations of HRQoL, dyspnea and symptoms that were not present prior to admission and a substantial proportion of these patients had not returned to work. Factors associated with a risk of poorer 12-month quality of life, may help to identify at-risk patients.

**Supplementary Information:**

The online version contains supplementary material available at 10.1186/s13613-022-00991-0.

## Introduction

The medium-term and long-term impact of COVID-19 on respiratory function and health-related quality of life are increasingly described [1–7]. However, most published studies have included patients with varying degrees of severity, have evaluated patients up to 6 months after the COVID-19 episode, and have not specifically focused on patients admitted to the intensive care unit (ICU) for a severe form of COVID-19. It is therefore unclear to what extent COVID-19 impacts health-related quality of life (HRQoL) and breathing comfort up to 12 months after ICU admission in this specific population, although sequelae are likely to be more pronounced in ICU survivors compared to patients who did not require ICU admission or who even did not require supplemental oxygen [1]. A better knowledge of these sequelae may help to improve the management of ICU survivors, which has consequences for all of society.

The high frequency and severity of sequelae, such as respiratory impairment and limb muscle weakness in patients with non-COVID-19-related acute respiratory distress syndrome (ARDS) [8–10] has stimulated rehabilitation programs that are still under development [11, 12]. Given the spread of the COVID-19 pandemic and the high proportion of patients requiring ICU admission, the rehabilitation community has called for specific action in relation to COVID-19 patients [13, 14], but very little information is currently available concerning the outcome of these programs [14].

The aim of the present study was to report temporal trends in dyspnea and HRQoL in COVID-19 patients admitted to the ICU for a severe form of COVID-19 and subsequently transferred to an inpatient rehabilitation unit. We also sought to identify the factors associated with alterations of respiratory comfort and HRQoL, including exercise capacity limitation and pulmonary function tests.

## Methods

### Study design

This study was conducted at La Pitié-Salpêtrière University Hospital in Paris, from 16 April 2020 to 25 June 2020. Patients admitted to an ICU of the great Paris area for acute respiratory failure with laboratory-confirmed SARS-CoV-2 infection and subsequently discharged and transferred to the inpatient respiratory rehabilitation facility were included. Laboratory-confirmed SARS-CoV-2 infection was defined as a positive result of real-time reverse transcriptase-polymerase chain reaction assay of nasal and pharyngeal swabs. Patients aged < 18 years were excluded. The study was approved by the French Pulmonary Medicine Society ethics committee (CEPRO No. 2020-070). Patients gave their informed consent. The study complied with the Strengthening the Reporting of Observational Studies in Epidemiology (STROBE) Statement guidelines (http://www.equator-network.org).

Rehabilitation was conducted in an inpatient unit with a multidisciplinary approach including oxygen and respiratory devices adaptation and education, nutritional support, psychiatric and psychologic evaluation and treatment, occupational therapist, especially for walking tools and plexopathy consequences, physiotherapists for neuromuscular electrostimulation. When possible, patients had stretching exercises and endurance training on treadmill or ergocycle, always with oxygen, twice a day.

### Data collection

Demographic data, medical history and data on the current episode of acute respiratory failure were abstracted from the patients' medical charts and electronic reports. These data included age, gender, body mass index, respiratory or cardiac comorbidity, Simplified Acute Physiologic Score (SAPS) 2 [15], oxygenation strategy, occurrence and severity of Acute Respiratory Distress Syndrome according to the Berlin definition [16], need for prone positioning, and organ support such as vasopressors, renal replacement therapy and extracorporeal lung support. Lengths of stay in the ICU, weaning unit (when applicable) and inpatient rehabilitation facility were also recorded.

Patients were evaluated in an outpatient clinic one to two months after ICU discharge. This evaluation included pulmonary function tests and arterial blood gases. Restrictive syndrome was defined as total lung capacity < 80% of predicted [17] obstructive syndrome was defined as forced expiratory volume in one second to forced vital capacity < 70%, diffusing capacity for carbon monoxide was considered to be abnormal when < 80% [18] and respiratory muscle weakness was defined as maximal inspiratory pressure < 80% [19]. A standardized six-minute walk test with continuous oximetry was performed. Walk distance was normalized to predicted distance, which was calculated based on age, sex, height and weight age, gender, height and weight [20]. The patient also completed a HRQoL assessment using the EQ-5D-3L [21] (EuroQol Research Foundation https://euroqol.org), which collates responses into five domains of HRQoL (mobility, self-care, usual activities, pain or discomfort, and anxiety or depression) with a three-level score (no problems, moderate problems, extreme problems or unable). EQ-5D-3L states were then converted into a single summary number or index value, which reflects how good or bad a health state is according to the preferences of the general population of a country or region. This approach ensures that the values represent the societal perspective. Here, we used the quality of life time trade-off utility values (EQ-5D-TTO), calculated using the French value set, with values generally ranging between 0 (death) to 1 (perfect health) [22]. Values less than 0 represent health states considered to be worse than death. Respondents were also asked to rate their perceived health on a visual analog scale (VAS) from 0 (worst) to 100 (best). Dyspnea was assessed by the modified Medical Research Council (mMRC) dyspnea scale [23] that grades the effect of breathlessness on daily activities from 0 “no limitations” to 4 “unable to leave home because of breathlessness”.

Twelve months after ICU discharge, patients were either evaluated in an outpatient clinic or contacted by telephone by a physician as a part of their post-ICU follow-up and a questionnaire including HRQoL assessment using the EQ-5D-3L and the mMRC dyspnea scale were administered and the patient's current weight was recorded. Patients were asked to list symptoms not present prior to COVID-19 (dichotomous outcome) such as: shoulder or arm weakness, persistent pain or dysesthesia, decreased range of motion of large joints, skin damage, especially in the neck, upper airway symptoms, including voice change and swallowing issues, anxiety or depression symptoms that needed medications. They were also asked whether or not they had returned to work. Pulmonary function tests and the six-minute walk test were performed at the physician's discretion, based on the patient's respiratory symptoms.

### Statistical analysis

The sample size was defined by the total number of patients admitted to the rehabilitation facility from an ICU, from the beginning to the end of the first wave of the COVID-19 epidemic in Paris, France. Quantitative variables were described as median (interquartile range) and qualitative variables were described as frequency (percentage).

Univariate linear regression models were used to identify factors associated with the six following dependent outcome variables: normalized 6-min walk test at 2 months, EQ-5D-3L (time trade-off and visual analog scale) at 2 months and 12 months and dyspnea mMRC at 12 months. Then, multivariate linear regression analysis were performed to identify factors indecently associated with these six dependent variables. All independent variables with *P* < 0.20 on univariate analysis were used to select the final model. We used a forward stepwise method based on Fisher Snedecor test for selection of the final model. Collinearity between variables and residuals were checked. Goodness of fit of the model was assessed with *R*^2^ and global *F*-test.

Statistical analyses were carried out using R v3.6.6 [24] (https://www.R-project.org/). *P* values ≤ 0.05 were considered to be statistically significant.

## Results

### Patients

Over the 3-month recruitment period, we enrolled 94 COVID-19 survivors, who were discharged from ICU and transferred to the inpatient rehabilitation facility (Table 1). Median age of these patients was 63 (49–70) years. Table 2 describes patient characteristics and management in the ICU. Among the 73 (78%) patients who were intubated, one (1%) developed mild ARDS, 42 (58%) developed moderate ARDS and 30 (41%) developed severe ARDS. Among intubated patients, 11 (15%) received extracorporeal lung support, and 24 (33%) were tracheostomized. Median ICU length of stay was 25 (15–46) days.

**Table 1:** Patient characteristics: factors associated with reduced health-related quality of life and dyspnea 12 months after intensive care unit admission on univariate regression analysis

	All patients n = 94	12-month EQ-5D-3L Visual Analog Scale n = 82		12-month EQ-5D-3L Time trade-off n = 86		12-month mMRC dyspnea scale n = 86	
		Linear regression coefficient ± SD	*P*	Linear regression coefficient ± SD	*P*	Linear regression coefficient ± SD	*P*
Age, *years, median (IQR)*	63 (49–70)	0.09 ± 0.14	0.540	0.01 ± 0.00	0.059	0.00 ± 0.01	0.915
Male gender*, n (%)*	67 (71)	7.27 ± 3.71	0.053	0.13 ± 0.07	0.071	− 0.20 ± 0.24	0.405
Body mass index, *kg.m*^*−2*^*, median (IQR)*	29.0 (26.3–33.6)	− 0.32 ± 0.25	0.216	− 0.01 ± 0.00	0.004	0.01 ± 0.02	0.413
Overweight*, n (%)*	39 (42)	− 2.11 ± 4.69	0.654	− 0.14 ± 0.09	0.120	0.00 ± 0.29	0.992
Obese*, n (%)*	39 (42)	− 3.56 ± 3.52	0.315	− 0.14 ± 0.07	0.042	0.15 ± 0.22	0.486
*Comorbidities*							
COPD, *n (%)*	7 (7)	2.67 ± 7.38	0.718	0.07 ± 0.14	0.596	0.50 ± 0.43	0.243
Asthma, *n (%)*	11 (12)	− 6.49 ± 5.13	0.210	− 0.11 ± 0.1	0.283	0.09 ± 0.33	0.795
Diabetes, *n (%)*	25 (27)	− 6.75 ± 3.92	0.089	0.01 ± 0.08	0.948	0.13 ± 0.25	0.596
Hypertension, *n (%)*	43 (46)	− 4.67 ± 3.5	0.186	0.01 ± 0.07	0.895	0.14 ± 0.22	0.541
Dyslipidemia, *n (%)*	24 (26)	1.94 ± 4.04	0.633	0.08 ± 0.08	0.294	0.05 ± 0.26	0.835
Active smoker, *n (%)*	32 (34)	− 8.81 ± 3.66	0.018	− 0.07 ± 0.07	0.319	0.46 ± 0.23	0.045
Chronic kidney disease, *n (%)*	8 (9)	− 5.5 ± 6.29	0.385	0.07 ± 0.12	0.537	− 0.22 ± 0.38	0.566
Immunosuppression, *n (%)*	12 (13)	− 0.44 ± 5.40	0.935	0.08 ± 0.10	0.442	− 0.12 ± 0.33	0.711
Charlson comorbidity index*, median (IQR)*	1 (0–2)	− 0.98 ± 0.93	0.297	0.01 ± 0.02	0.620	0.02 ± 0.06	0.746

Continuous variables are expressed as median (interquartile range [IQR]) and categorical variables are expressed as absolute value (%)

Health-related quality of life is assessed with the EQ-5D-3L (EuroQol Research Foundation https://euroqol.org). Quality of life time trade-off utility values were calculated using the French value set. Perceived health was rated on a visual analog scale (VAS) from 0 (worst) to 100 (best). Dyspnea was assessed by the modified Medical Research Council (mMRC) dyspnea scale

mMRC, modified Medical Research Council dyspnea scale; COPD, chronic obstructive pulmonary disease

The linear regression coefficients represent the average increase or decrease in the variable to be explained when we compare two subjects with explanatory quantitative variables that differ by one unit or when we compare two subjects with explanatory qualitative variables taking the reference value for one of the subjects and another value for the second subject

**Table 2:** Intensive care unit (ICU) and inpatient rehabilitation unit stay: factors associated with reduced health-related quality of life and dyspnea 12 months after ICU admission on univariate regression analysis

	All patients *n* = 94	12-month EQ-5D-3L Visual Analog Scale *n* = 82		12-month EQ-5D-3L Time trade-off *n* = 86		12-month mMRC dyspnea scale *n* = 86	
		Linear regression coefficient ± SD	*P*	Linear regression coefficient ± SD	*P*	Linear regression coefficient ± SD	*P*
ICU stay							
PaO_2_/FiO_2_ on ICU admission, *mmHg, median (IQR)*	136 (109–185)	0.02 ± 0.03	0.402	0.001 ± 0.001	0.292	− 0.001 ± 0.002	0.931
SAPS 2*, median (IQR)*	33 (26–39)	0.04 ± 0.17	0.794	− 0.001 ± 0.003	0.714	0.001 ± 0.011	0.900
*Oxygenation strategy*			0.275		0.860		0.969
Standard oxygen, *n (%)*	61 (65)	-		-		-	
HFNC, *n (%)*	10 (11)	− 9.76 ± 6.34	0.128	0.04 ± 0.12	0.736	0.06 ± 0.37	0.869
CPAP, *n (%)*	17 (18)	− 6.55 ± 4.73	0.170	− 0.003 ± 0.094	0.977	0.11 ± 0.30	0.725
NIV, *n (%)*	6 (6)	1.07 ± 6.80	0.875	0.15 ± 0.14	0.270	0.17 ± 0.44	0.698
Worst PaO_2_/FiO_2_ in the ICU, *mmHg, median (IQR)*	112 (85–145)	0.04 ± 0.03	0.275	0.001 ± 0.001	0.120	− 0.003 ± 0.002	0.120
*Intubation, n (%)*	73 (78)	− 2.72 ± 4.69	0.564	− 0.13 ± 0.09	0.141	− 0.05 ± 0.28	0.866
Neuromuscular blocking agents*, n (%)*	61 (65)	− 1.88 ± 5.24	0.722	0.07 ± 0.11	0.543	− 0.53 ± 0.33	0.107
Prone positioning*, n (%)*	59 (63)	− 2.80 ± 3.75	0.457	− 0.07 ± 0.07	0.340	− 0.25 ± 0.23	0.275
Vasopressors*, n (%)*	25 (27)	− 3.87 ± 3.87	0.320	0.01 ± 0.08	0.924	− 0.11 ± 0.24	0.644
Renal replacement therapy*, n (%)*	8 (9)	2.48 ± 5.95	0.678	0.05 ± 0.12	0.680	0.06 ± 0.38	0.879
Extracorporeal lung support*, n (%)*	11 (12)	1.44 ± 5.25	0.784	− 0.08 ± 0.11	0.476	0.42 ± 0.33	0.201
Duration of mechanical ventilation*, days*	30 (20–41)	0.04 ± 0.12	0.729	− 0.003 ± 0.003	0.211	0.01 ± 0.01	0.379
Tracheostomy*, n (%)*	24 (26)	− 4.02 ± 3.91	0.307	− 0.25 ± 0.07	0.001	0.26 ± 0.25	0.288
Steroids given in the ICU*, n (%)*	15 (16)	− 2.41 ± 4.99	0.631	− 0.01 ± 0.10	0.905	0.13 ± 0.31	0.674
ICU length of stay, *days, median (IQR)*	25 (15–46)	− 0.04 ± 0.10	0.685	− 0.005 ± 0.002	0.005	0.01 ± 0.01	0.069
**On admission to the rehabilitation facility**							
mMRC dyspnea scale*, median (IQR)*	3 (2–4)	− 3.37 ± 1.54	0.032	− 0.04 ± 0.03	0.151	0.09 ± 0.09	0.331
Weight loss, %	11 (7–15)	0.41 ± 0.28	0.156	0.01 ± 0.01	0.128	− 0.03 ± 0.02	0.124
Albumin, *g.L*^*−1*^*, median (IQR)*	29 (26–31)	0.22 ± 0.34	0.522	0.01 ± 0.01	0.852	0.01 ± 0.02	0.721
Prealbumin, *g.L*^*−1*^*, median (IQR)*	0.24 (0.19–0.28)	6.00 ± 21.02	0.776	− 0.10 ± 0.42	0.804	− 1.86 ± 1.29	0.153

Continuous variables are expressed as median (interquartile range [IQR]) and categorical variables are expressed as absolute value (%)

Health-related quality of life is assessed with the EQ-5D-3L (EuroQol Research Foundation https://euroqol.org). Quality of life time trade-off utility values were calculated using the French value set. Perceived health was rated on a visual analog scale (VAS) from 0 (worst) to 100 (best). Dyspnea was assessed by the modified Medical Research Council (mMRC) dyspnea scale

SAPS, Simplified Acute Physiologic Score; HFNC, high-flow nasal cannula; CPAP, continuous positive airway pressure; NIV, noninvasive ventilation; mMRC, modified Medical Research Council dyspnea scale

The linear regression coefficients represent the average increase or decrease in the variable to be explained when we compare two subjects with explanatory quantitative variables that differ by one unit or when we compare two subjects with explanatory qualitative variables taking the reference value for one of the subjects and another value for the second subject

**Fig. 1:**
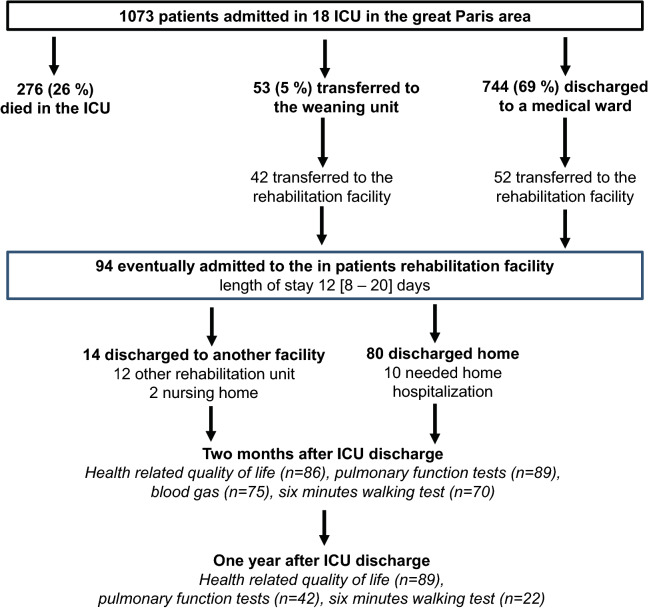
Study flowchart. ICU, intensive care unit. Home hospitalization is defined as a service providing home-based, short-term complex interventions aiming at substituting conventional hospitalization

Figure 1 displays the patient flowchart. Patients came from 18 ICUs of the great Paris area (1073 admission over the study period, 26% mortality). Among the 744 patients who were discharges to a medical ward, 52 (55%) were transferred directly to the inpatient rehabilitation facility. In addition 42 patients (45%) were first transferred to a weaning unit (median length of stay: 12 [9–16] days) and then subsequently transferred to the inpatient rehabilitation facility. The median duration of mechanical ventilation was 30 (20–41) days.

On admission to the inpatient rehabilitation facility, the mMRC dyspnea scale was 3 (2–4) and weight loss was 11 (7–15) %. Patients spent 12 (8–20) days in the inpatient rehabilitation facility (Table 2). After discharge from the inpatient rehabilitation facility, 14 (15%) patients were transferred to another facility (12 to another rehabilitation unit and 2 to a nursing home) and 80 (85%) were discharged home, including 10 patients who required home hospitalization. No patient died or was transferred to an ICU. Overall, patients spent 50 (35–74) days in hospital (Fig. 1).

### Two-month follow-up assessment

The real follow-up at the time of the 2-month visit was 43 (34–55) days after ICU discharge.

**Fig. 2:**
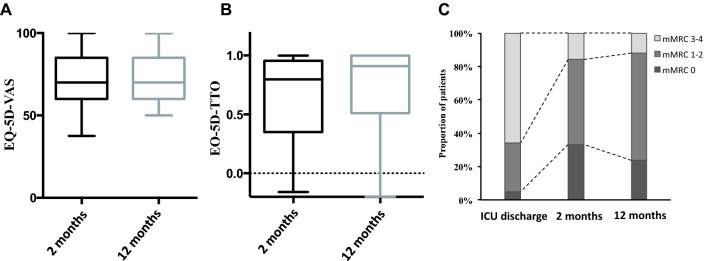
Temporal changes in health-related quality of life (panel A and B) and dyspnea (Panel C) 2 months and 12 months after intensive care unit (ICU) discharge for severe COVID-19. EQ-5D VAS (panel A): patients rated their perceived health on a visual scale from 0 (worst) to 100 (best) called visual analog scale. EQ-5D TTO (panel B): Quality of life “time trade-off” utility values were calculated using the French algorithm with values generally ranging between 0 (death) to 1 (perfect health) [22]. mMRC dyspnea scale (panel C): modified Medical Research Council dyspnea scale. EQ-5D VAS and EQ-5D TTO are represented as box and whiskers where the red center line denotes the median value (50th percentile), the box contains the 25th to 75th percentiles and the whiskers mark the 5th and 95th percentiles

Two months after ICU admission, patients who survived COVID-19 were 9 (5–15) % below their baseline body weight. At the 2-month visit, 12 (13%) patients were still receiving supplemental oxygen. The mMRC dyspnea scale was 1 (0–2) (*P* < 0.001 vs. ICU discharge) (Fig. 2). Pulmonary function tests were performed in 89 (95%) patients and arterial blood gases were determined in 75 (80%) patients (Table 3). Briefly, 37 (47%) patients had a restrictive syndrome, 9 (10%) had an obstructive syndrome, 54 (69%) had a decreased diffusing capacity for carbon monoxide and 44 (56%) had respiratory muscle weakness. Twenty-four (26%) patients were unable to perform 6-min walk test due to obvious muscle weakness. 6-min walk test was 392 m (322–484), which represented 58 (47–69) % of predicted normal value.

**Table 3:** Pulmonary function tests and arterial blood gases at the 2-month assessment: factors associated with reduced health-related quality of life and dyspnea 12 months after intensive care unit admission on univariate regression analysis

	All patients *n* = 94	12-month EQ-5D-3L Visual Analog Scale *n* = 82		12-month EQ-5D-3L Time trade-off *n* = 86		12-month mMRC dyspnea scale *n *= 86	
		Linear regression coefficient ± SD	*p*	Linear regression coefficient ± SD	*p*	Linear regression coefficient ± SD	*p*
At two-month assessment							
mMRC dyspnea scale*, median (IQR)*	1 (0–2)	− 2.39 ± 1.62	0.146	− 0.06 ± 0.03	0.059	0.25 ± 0.09	0.012
EQ-5D-3L, Visual analog scale*, median (IQR)*	70 (60–85)	0.42 ± 0.10	< 0.001	0.01 ± 0.00	0.001	0.05 ± 0.52	0.917
EQ-5D-3L, time trade-off*, median (IQR)*	0.80 (0.36–0.91)	15.29 ± 5.60	0.008	0.33 ± 0.10	0.002	− 1.09 ± 0.33	0.002
Six-minute walk test*, m, median (IQR)*	392 (322–484)	0.01 ± 0.02	0.952	0.0004 ± 0.0004	0.326	− 0.001 ± 0.001	0.459
Normalized six-minute walk test*, %, median (IQR)*	58 (47–69) %	0.09 ± 0.12	0.437	0.004 ± 0.002	0.092	− 0.01 ± 0.01	0.171
Total lung capacity^a^*, % of predicted, median (IQR)*	78 (63–89)	− 0.57 ± 0.12	0.632	− 0.001 ± 0.007	0.872	− 0.01 ± 0.01	0.175
Total lung capacity^a^ < 80% of predicted, *n (%)*	37 (47)	− 2.04 ± 3.86	0.599	− 0.02 ± 0.07	0.799	− 0.32 ± 0.23	0.04
Forced vital capacity*, % of predicted, median (IQR)*	76 (67–93)	0.02 ± 0.09	0.832	0.002 ± 0.002	0.901	− 0.02 ± 0.01	0.114
Forced vital capacity, < 80% of predicted, *n (%)*	39 (45)	4.14 ± 3.59	0.253	0.02 ± 0.07	0.736	− 0.47 ± 0.22	0.037
Forced expiratory volume in one second*, % of predicted, median (IQR)*	77 (67–90)	0.05 ± 0.09	0.572	0.001 ± 0.002	0.704	− 0.01 ± 0.01	0.704
FEV_1_/FVC*, %, median (IQR)*	83 (77–87)	0.10 ± 0.19	0.598	0.001 ± 0.004	0.681	− 0.02 ± 0.01	0.681
FEV_1_/FVC < 70%*, n (%)*	9 (10)	− 6.31 ± 6.12	0.305	− 0.05 ± 0.12	0.678	− 0.05 ± 0.12	0.678
Diffusing capacity for carbon monoxide^b^, *% of predicted, median (IQR)*	56 (45–67)	0.08 ± 0.12	0.528	0.002 ± 0.002	0.433	− 0.01 ± 0.01	0.433
Carbon monoxide transfer coefficient^b^, *% of predicted, median (IQR)*	87 (76–96)	0.11 ± 0.11	0.279	0.003 ± 0.002	0.158	− 0.01 ± 0.01	0.158
Diffusing capacity for carbon monoxide^b^ < 80% of predicted, *n (%)*	54 (69)	− 0.23 ± 4.28	0.958	− 0.01 ± 0.08	0.895	0.03 ± 0.26	0.895
Sniff nasal inspiratory pressure^c^, *% of predicted, median (IQR)*	60 (43–82)	− 0.02 ± 0.07	0.818	0.002 ± 0.001	0.909	− 0.002 ± 0.005	0.909
Maximal inspiratory pressure^d^, *% of predicted, median (IQR)*	78 (59–95)	− 0.04 ± 0.05	0.491	0.001 ± 0.001	0.523	− 0.001 ± 0.004	0.523
Maximal inspiratory pressure < 80% of predicted, *n (%)*	44 (56)	− 0.24 ± 3.71	0.949	0.05 ± 0.07	0.487	− 0.05 ± 0.07	0.487
PaO_2_^e^, *mmHg, median (IQR)*	91 (82–97)	0.27 ± 0.18	0.140	0.00 6 ± 0.003	0.081	− 0.03 ± 0.01	0.002
PaCO_2_^e^, *mmHg, median (IQR)*	38 (35–40)	− 0.99 ± 0.53	0.065	− 0.01 ± 0.01	0.248	0.06 ± 0.03	0.093
pH^e^*, median (IQR)*	7.44 (7.42–7.46)	70.33 ± 69.77	0.318	− 0.15 ± 1.17	0.897	− 4.35 ± 4.17	0.300
SaO_2_^e^, *%, median (IQR)*	97 (96–98)	2.79 ± 1.36	0.044	0.04 ± 0.02	0.105	− 0.28 ± 0.08	0.001

Continuous variables are expressed as median (interquartile range [IQR]) and categorical variables are expressed as absolute value (%)

Health-related quality of life is assessed with the EQ-5D-3L (EuroQol Research Foundation https://euroqol.org). Quality of life time trade-off utility values were calculated using the French value set. Perceived health was rated on a visual analog scale (VAS) from 0 (worst) to 100 (best). Dyspnea was assessed by the modified Medical Research Council (mMRC) dyspnea scale

mMRC, modified Medical Research Council dyspnea scale; FEV_1_, forced expiratory volume in one second; FVC, forced vital capacity

^a^Data available for 78 cases, ^b^Data available for 82 cases, ^c^Data available for 76 cases, ^d^Data available for 81 cases, ^e^Data available for 75 cases

The linear regression coefficients represent the average increase or decrease in the variable to be explained when we compare two subjects with explanatory quantitative variables that differ by one unit or when we compare two subjects with explanatory qualitative variables taking the reference value for one of the subjects and another value for the second subject

Seventy-seven patients (82%) completed the five questions of the EQ-5D-3L, 69 (73%) patients completed the EQ-5D-3L VAS and 61 (65%) completed both instruments. Table 4 shows the answers to the EQ-5D-3L with a sum of 7 (5–9) and 0.80 (0.36–0.91) after normalization to the French population using the specific TTO value set for France [25] (Fig. 2). Finally, the EQ-5D VAS was 70 (60–85).

**Table 4:** EuroQol questionnaire (EQ-5D-3L) at the 2-month and 12-month assessments

Variable	2-month assessment *n* = *77*	12-month assessment *n* = *86*	*P*
*Mobility*			0.530
I have no problems with walking about, *n (%)*	52 (68)	61 (71)	
I have some problems with walking about, *n (%)*	25 (33)	25 (29)	
I am confined to bed, *n (%)*	0 (0)	0 (0)	
*Self-care*			0.413
I have no problems with self-care, *n (%)*	65 (84)	68 (79)	
I have some problems with washing or dressing myself, *n (%)*	10 (13)	16 (19)	
I am unable to wash or dress myself, *n (%)*	2 (3)	2 (2)	
*Usual Activities*			0.074
I have no problems performing my usual activities, *n (%)*	43 (56)	65 (76)	
I have some problems performing my usual activities, *n (%)*	12 (16)	17 (20)	
I am unable to perform my usual activities, *n (%)*	22 (29)	4 (5)	
*Pain Discomfort*			0.457
I have no pain or discomfort, *n (%)*	40 (52)	57 (66)	
I have moderate pain or discomfort, *n (%)*	29 (38)	23 (27)	
I have extreme pain or discomfort, *n (%)*	8 (10)	6 (7)	
*Anxiety depression*			0.494
I am not anxious or depressed, *n (%)*	54 (70)	46 (53)	
I am moderately anxious or depressed, *n (%)*	16 (21)	32 (37)	
I am extremely anxious or depressed, *n (%)*	7 (9)	8 (9)	
*EQ-5D-3L, sum, median, IQR*	7 (5–9)	6 (5–8)	
*EQ-5D-3L, time trade-Off, France, median, IQR*	0.80 (0.36–0.91)	0.91 (0.52–1.00)	0.012
*EQ-5D-3L, Visual Analog Scale, median, IQR*	70 (60–85)	70 (60–85)	0.114

Continuous variables are expressed as median (interquartile range [IQR]) and categorical variables are expressed as absolute value (%)

In Additional file 1: Tables S1, S2, S3 show the factors associated with reduced normalized six-minute walking distance and reduced HRQoL identified on univariate analysis. On multivariate analysis, the only factor associated with a reduced normalized six-minute walk test was length of stay prior to rehabilitation unit admission (linear regression coefficient − 2.14, 95% confidence interval [95% CI] − 3.24 to − 1.03, *P* < 0.001) (p *F*-statistic < 0.001, R2 = 0.17). Factors independently associated with EQ-5D-3L TTO were male gender (linear regression coefficient 0.26, 95% CI 0.11–0.42, *P* = 0.001) and immunosuppression (linear regression coefficient 0.23, 95%CI 0.02–0.44,* P* = 0.031) (p F-statistic < 0.001, R2 = 0.20). The only factor independently associated with EQ-5D-3L VAS was dyspnea on admission to the inpatient rehabilitation facility (linear regression coefficient − 5.31, 95%CI − 8.86 – − 1.77, *P* = 0.005) (p F-statistic = 0.005, R2 = 0.12). On multivariate analysis, pulmonary function tests were not associated with six-minute walking distance and reduced HRQoL.

### 12-month follow-up assessment

The 12-month follow-up was performed in 89 (91%) patients. Four patients were lost to follow-up and one patient died as the result of a car accident. The real follow-up at the time of the 12-month visit was 11.9 (11.3–12.2) months. The 12-month assessment was performed at the outpatient clinic in 53 patients (60%) and by phone only in the remaining 36 patients (40%).

**Fig. 3:**
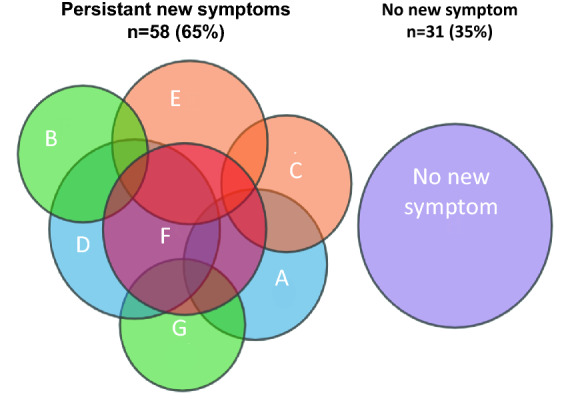
Representation of symptoms not present before COVID-19 in the 89 patients assessed 12 months after intensive care unit admission. Numbers represent patients with symptoms: **A** plexopathy; (*n* = 17);** B** persistent pain or dysesthesia (*n* = 14);** C** decreased range of motion of large joints, mostly the shoulders (*n* = 14); **D** altered appearance of the skin of the neck (*n* = 24);** E** upper airway symptoms (*n* = 20);** F** anxiety or depression requiring medication (*n* = 22); **G** reported “slow thinking” (*n* = 13). 31 patients did not report any of these symptoms

Twelve months after ICU admission, patients who survived COVID-19 were 3 (− 1 to 7) % below their baseline body weight. A plexopathy was present in 17 (19%) patients, confirmed by an electromyography. Fourteen (16%) patients reported persistent pain or dysesthesia, 14 (16%) patients reported decreased range of motion of large joints (mostly the shoulders), 24 (27%) patients reported an altered appearance of their neck skin and 20 (22%) patients reported upper airway symptoms, including voice change. Twenty-two (25%) patients were receiving medications for anxiety or depression symptoms. Thirteen patients (15%) reported “slow thinking”. Overall, 58 (65%) patients reported at least one symptom not present prior to ICU admission (Fig. 3). Among the 44 patients who were working prior to ICU admission, 27 (61%) had returned to work (12 [44%] part-time).

The mMRC dyspnea scale was 1 (1–2) (*p* = 0.809 vs. 2-month assessment). Pulmonary function tests were performed in 42 (45%) patients (Aditional file 1: Table S4). Forced vital capacity and diffusing capacity for carbon monoxide improved. Six-minute walk test was 506 m (440–562) (*p* < 0.001 vs. 2-month assessment), which represented 80 (72–82) % of predicted normal value (*p* < 0.001 vs. 2-month assessment).

Eighty-six patients (90%) completed the five questions of the EQ-5D-3L, 82 (85%) patients completed the EQ-5D-3L VAS and 86 (90%) completed both instruments. Table 4 shows the answers to the EQ-5D-3L with a sum of 6 (5–8) and 0.91 (0.52–1.00) after normalization to the French population (*p* = 0.12 vs. 2-month assessment) [25]. Finally, the score on the EQ-5D analog scale was 70 (60–85) (*p* = 0.114 vs. 2-month assessment).

Tables 1, 2 and 3 show the factors associated with dyspnea and HRQoL identified on univariate analysis. On multivariate analysis, factors associated with a EQ-5D-3L TTO were body mass index on ICU admission (linear regression coefficient − 0.01, 95%CI − 0.02–0.00, *p* = 0.005) and tracheostomy (linear regression coefficient  − 0.22, 95%CI  − 0.36 to − 0.08, *p* = 0.002) (*p* F-statistic < 0.001, *R*2 = 0.20). Factors independently associated with EQ-5D-3L VAS were male gender (linear regression coefficient  − 8.74, 95%CI 1.37–16.11, *p* = 0.023) and active smoking (linear regression coefficient  − 3.00, 95%CI − 5.88–0.12, *p* = 0.044) (*p* *F*-statistic < 0.001, *R*2 = 0.20). The only factor independently associated with mMRC dyspnea scale was active smoking (linear regression coefficient 0.50, 95%CI 0.04–0.95, *p* = 0.036) (*p* F-statistic = 0.036, *R*2 = 0.056). On multivariate analysis, 2-month pulmonary function tests were not associated with 12-month HRQoL or dyspnea mMRC.

## Discussion

The main and major findings of our study are as follows. In patients who survived a severe form of COVID-19 that had required ICU admission and were subsequently transferred to an inpatient rehabilitation facility: (1) HRQoL was still altered 12 months after ICU admission, with a substantial proportion of patients reporting at least one symptom related to the ICU stay; (2) some variables may predict earlier the alteration of health-related quality of life.

Because the survival of patients with ARDS has dramatically improved, the morbidity and sequelae in survivors have now become a major subject of interest. Over the past 20 years, several investigators have evaluated morbidity among survivors and have reported alteration of lung function, exercise capacity and HRQoL after ICU discharge [8, 9, 26–28]. To date, most data available for COVID-19 patients who required ICU admission come from 2 months and up to 6 months follow-up [1, 4–7]. These data are consistent with our results, showing similar alterations of six-minute walking test, pulmonary function tests and exercise capacity. Data on 12 months follow-up are scarce and, to date, no study has been devoted to ICU patient [4]. In contrast, in our study, all patients were admitted to the ICU and 78% of them were intubated. It is not clear to what extent COVID-19 will leave sequelae, such as persistent limitations in respiratory, physical, and functional outcomes, but such sequelae are likely to be more pronounced in the subgroup of ICU survivors [14, 29]. A better knowledge of these sequelae may help to improve the management of ICU survivors.

A strikingly high proportion of patients reported at least one symptom that was not present prior to ICU admission. This proportion seems to be higher than that reported in a previous cohort of non-COVID ARDS patients with a similar ICU length of stay and a high proportion of tracheostomized patients [8]. This difference could be partly explained by the high rate of prone positioning that is likely to alter the skin and cause brachial plexus injuries, although factors directly related to SARS-CoV-2 infection cannot be ruled out. There are a number of differences between SARS-CoV-2 and other infectious agents that target the lungs, such as the intense proinflammatory cytokine storm and marked endothelial dysfunction [30]. Finally, 12 months after ICU admission, HRQoL had not returned to normal, as EQ-5D visual analog scale and TTO were below the normal range of the French population [22]. While pulmonary function tests at 2 months were moderately, but not dramatically altered, and given the fact that a continuous improvement over the first year has been reported [1, 4] altered lung function may not explain the decreased HRQoL. On the other hand, the persistence of symptoms that were not present prior to ICU admission might contribute to this decreased HRQoL.

A previous study in COVID-19 patients showed improvement of dyspnea and exercise capacity between two months and 6 months [4], while we did not observe any change in dyspnea and HRQoL between these two time-points. In our cohort, dyspnea improved between ICU discharge and 2-month, but not thereafter, which could be explained by the fact that all patients in our cohort were rehabilitated, which may have rapidly improved dyspnea after ICU discharge.

Identifying, as early as possible, those patients at risk of poor long-term quality of life constitutes a major challenge, as, after discharge from the rehabilitation unit, these patients could integrate an outpatient rehabilitation program in order to improve their 12-month quality of life as much as possible [14]. Unfortunately, few factors in our cohort were independently associated with poorer 12-month quality of life. However, obese patients, active smokers, tracheostomized patients and patients with a long ICU length of stay were at higher risk of poorer HRQoL at 12 months. Surprisingly, markers of severity such as hypoxemia and SAPS 2 were not associated with 12-month HRQoL.

This study has several limitations. First, we limited the 12-month evaluation to dyspnea and HRQoL and did not perform pulmonary function tests and six-minute walk test in all patients. These tests were requested by the patient’s physician, based on his/her clinical judgment. However, as these tests were performed in only very few patients, and exclusively in those who remained severely symptomatic, we did not report the results of these tests. Second, this study did not comprise a control group, which precludes comparison of the prevalence and severity of symptoms with patients admitted to the ICU for acute respiratory failure due to a cause other than COVID-19. Third, because patients were admitted during the first wave of the epidemic, many did not receive corticosteroids, which are now an integral part of the treatment of severe forms of COVID-19. Fourth, all patients were admitted to the inpatient rehabilitation facility after discharge from ICU, which precludes assessment of the impact of rehabilitation on 12-month HRQoL. Last, evaluation were performed by phone, which is less reliable than in an outpatient clinics.

### Conclusion

Twelve months after ICU admission for COVID-19 and subsequent rehabilitation, a substantial proportion of patients reported alterations of HRQoL, dyspnea and symptoms that were not present prior to admission and a substantial proportion of these patients had not returned to work. Our study identifies factors associated with a risk of poorer 12-month quality of life, which may help to identify at-risk patients. Our findings highlight the importance of follow-up of patients who have experienced severe forms of COVID-19. Further studies are necessary to determine whether an early outpatient rehabilitation program after discharge from an inpatient rehabilitation facility can help to improve one-year HRQoL in at-risk patients. Longer follow-up is also necessary.

## Supplementary Information

**Additional file 1: Tables S1, S2, S3.** Factors associated with reduced six-minute walking distance and reduced HRQoL two months after intensive care unit admission identified on univariate analysis.

## Data Availability

The datasets used and/or analyzed during the current study are available from the corresponding author on reasonable request.

## Abbreviations

COVID-19: Coronavirus disease 2019
ICU: Intensive care unit
HRQoL: Health-related quality of life
ARDS: Acute respiratory distress syndrome
SARS-CoV-2: Severe acute respiratory syndrome coronavirus 2
SAPS: Simplified Acute Physiologic Score
mMRC: Modified Medical Research Council dyspnea scale
COPD: Chronic obstructive pulmonary disease
HFNC: High-flow nasal cannula
NIV: Non-invasive ventilation
CPAP: Continuous positive airway pressure
