# Supplementary material for: Health-related quality of life of COVID-19 two and 12 months after intensive care unit admission
Source: Ann Intensive Care. 2022 Feb 20;12:16. doi: 10.1186/s13613-022-00991-0 (PMC8858438; doi:10.1186/s13613-022-00991-0)
Supplement: Supplementary file 1 — Additional file 1: Tables S1, S2, S3. Factors associated with reduced six-minute walking distance and reduced HRQoL two months after intensive care unit admission identified on univariate analysis. [file 13613_2022_991_MOESM1_ESM.docx]

**Online Supplement**

**Health-related quality of life of COVID-19 two and twelve months after intensive care unit admission**

Alexandre Demoule, Elise Morawiec, Maxens Decavele, Raphaelle Ohayon, Roxane Malrain, Maria Alejandra Galarza, Christian Strauss, Capucine Morelot-Panzini, Thomas Similowski, Yann De Rycke, Jesus Gonzalez-Bermejo

**Table E1. Patient characteristics: factors associated with reduced exercise capacity and health-related quality of life two months after intensive care unit admission on univariate analysis**

**Table E2. Intensive care unit (ICU) and rehabilitation unit stay: factors associated with reduced exercise capacity and health-related quality of life two months after intensive care unit admission on univariate analysis**

**Table E3. Dyspnea, pulmonary function tests and arterial blood gases at the 2-month assessment: factors associated with reduced exercise capacity and health-related quality of life two months after intensive care unit admission on univariate analysis**

**Table E1. Patients characteristics: factors associated with reduced exercise capacity and health-related quality of life two months after intensive care unit admission**

|  | **Two-month**  **Six-minute walk test**  **% predicted**  **n=70** | | **Two -month EQ-5D-3L**  **Visual Analog Scale**  **n=69** | | **Two-month EQ-5D-3L**  **Time Trade-Off**  **n=77** | |
| --- | --- | --- | --- | --- | --- | --- |
|  | Linear regression coefficient ± SD | **P** | Linear regression coefficient ± SD | **P** | Linear regression coefficient ± SD | **P** |
| Age, *years, median (IQR)* | -0.01 ± 0.16 | 0.964 | 0.24 ± 0.18 | 0.184 | 0.02 ± 0.01 | 0.535 |
| Male gender*, n (%)* | -1.05 ± 4.62 | 0.822 | 3.01 ± 5.27 | 0.570 | 0.29 ± 0.07 | 0.001 |
| Body mass index, *kg.m^-2^, median (IQR)* | 0.13 ± 0.31 | 0.667 | -0.19 ± 0.35 | 0.578 | -0.02 ± 0.01 | 0.001 |
| Overweight*, n (%)* | 0.54 ± 5.59 | 0.923 | 0.26 ± 6.11 | 0.966 | -0.21 ± 0.12 | 0.049 |
| Obese*, n (%)* | 5.29 ± 3.89 | 0.179 | 1.17 ± 4.59 | 0.800 | -0.18 ± 0.08 | 0.025 |
| *Comorbidities* |  |  |  |  |  |  |
| COPD, *n (%)* | 0.22 ± 7.71 | 0.978 | 21.09 ± 8.19 | 0.012 | -0.12 ± 0.18 | 0.507 |
| Asthma, *n (%)* | 0.08 ± 5.92 | 0.990 | -14.79 ± 7.17 | 0.042 | -0.09 ± 0.11 | 0.377 |
| Diabetes, *n (%)* | -0.04 ± 4.53 | 0.994 | -8.71 ± 4.96 | 0.084 | -0.02 ± 0.09 | 0.446 |
| Hypertension, *n (%)* | 4.43 ± 3.93 | 0.264 | -1.93 ± 4.53 | 0.671 | -0.02 ± 0.09 | 0.446 |
| Dyslipidemia, *n (%)* | -1.61 ± 4.45 | 0.719 | -0.96 ± 5.07 | 0.849 | 0.03 ± 0.09 | 0.758 |
| Active smoker, *n (%)* | 0.57 ± 4.13 | 0.891 | -1.62 ± 4.67 | 0.729 | 0.09 ± 0.08 | 0.268 |
| Chronic kidney disease, *n (%)* | 10.09 ± 9.71 | 0.303 | -7.64 ± 7.32 | 0.301 | 0.05 ± 0.15 | 0.731 |
| Immunosuppression, *n (%)* | -10.34 ± 6.49 | 0.116 | -6.25 ± 6.57 | 0.345 | 0.25 ± 0.11 | 0.028 |
| Charlson comorbidity index*, median (IQR)* | -0.78 ± 1.05 | 0.458 | -1.59 ± 1.09 | 0.149 | -0.01 ± 0.03 | 0.828 |

Health related quality of life is assessed with the EQ-5D-3L (EuroQol Research Foundation https://euroqol.org). Quality of life time trade-off utility values were calculated using the French value set. Perceived health was rated on a visual analog scale (VAS) from 0 (worst) to 100 (best). Dyspnea was assessed by the modified Medical Research Council (mMRC) dyspnea scale.

COPD, chronic obstructive pulmonary disease.

The linear regression coefficients represent the average increase or decrease in the variable to be explained when we compare two subjects with explanatory quantitative variables that differ by one unit or when we compare two subjects with explanatory qualitative variables taking the reference value for one of the subjects and another value for the second subject.

**Table E2. Intensive care unit (ICU) and rehabilitation unit stay: factors associated with reduced exercise capacity and health-related quality of life two months after intensive care unit admission**

|  | **Two-month**  **Six-minute walk test**  **% predicted**  **n=70** | | **Two-month EQ-5D-3L**  **Visual Analog Scale**  **n=69** | | **Two-month EQ-5D-3L**  **Time Trade-Off**  **n=77** | |
| --- | --- | --- | --- | --- | --- | --- |
|  | Linear regression coefficient ± SD | **P** | Linear regression coefficient ± SD | **P** | Linear regression coefficient ± SD | **P** |
| **ICU stay** |  |  |  |  |  |  |
| PaO_2_/FiO_2_ on ICU admission, *mmHg, median (IQR)* | 0.04 ± 0.03 | 0.163 | 0.02 ± 0.04 | 0.530 | 0.01 ± 0.01 | 0.706 |
| SAPS 2 | -0.06 ± 0.18 | 0.745 | -0.09 ± 0.24 | 0.712 | 0.01 ± 0.01 | 0.183 |
| *Oxygenation strategy* |  | 0.848 |  |  |  |  |
| Standard oxygen, *n (%)* |  |  |  |  |  |  |
| HFNC, *n (%)* | 4.50 ± 5.27 | 0.396 | -0.67 ± 7.58 | 0.930 | 0.07 ± 0.13 | 0.596 |
| CPAP, *n (%)* | 2.20 ± 7.27 | 0.764 | 5.20 ± 5.88 | 0.380 | 0.02 ± 0.10 | 0.826 |
| NIV, *n (%)* | 2.73 ± 7.27 | 0.708 | -4.95 ± 8.10 | 0.543 | 0.27 ± 0.16 | 0.088 |
| Worst PaO_2_/FiO_2_ in the ICU, *mmHg, median (IQR)* | 0.04 ± 0.04 | 0.269 | 0.03 ± 0.04 | 0.443 | 0.01 ± 0.01 | 0.141 |
| *Intubation, n (%)* | -8.07 ± 4.62 | 0.085 | -7.27 ± 4.83 | 0.137 | -0.07 ± 0.09 | 0.474 |
| Neuromuscular blocking agents*, n (%)* | 0.22 ± 6.34 | 0.972 | 3.98 ± 6.89 | 0.567 | 0.03 ± 0.12 | 0.791 |
| Prone positioning*, n (%)* | -10.76 ± 4.05 | 0.010 | -12.08 ± 4.31 | 0.007 | -0.08 ± 0.08 | 0.302 |
| Vasopressors*, n (%)* | 1.81 ± 4.86 | 0.710 | -4.42 ± 5.43 | 0.419 | -0.13 ± 0.08 | 0.123 |
| Renal replacement therapy*, n (%)* | -7.50 ± 7.64 | 0.330 | 6.00 ± 8.56 | 0.486 | -0.13 ± 0.13 | 0.290 |
| Extracorporeal lung support*, n (%)* | -3.87 ± 6.02 | 0.523 | 1.62 ± 7.30 | 0.826 | -0.01 ± 0.12 | 0.995 |
| Duration of mechanical ventilation*, days* | -0.34 ± 0.12 | 0.006 | -0.08 ± 0.15 | 0.573 | 0.01 ± 0.01 | 0.859 |
| Tracheostomy*, n (%)* | 2.64 ± 4.31 | 0.542 | -0.56 ± 5.42 | 0.918 | -0.02 ± 0.11 | 0.868 |
| Steroids given in the ICU*, n (%)* | -5.64 ± 5.40 | 0.300 | -16.88 ± 5.98 | 0.006 | 0.09 ± 0.11 | 0.403 |
| ICU length of stay, *days, median (IQR)* | -0.36 ± 0.10 | 0.000 | -0.25 ± 0.12 | 0.049 | -0.03 ± 0.01 | 0.186 |
| **On admission to the rehabilitation facility** |  |  |  |  |  |  |
| mMRC dyspnea scale*, median (IQR)* | -1.94 ± 1.69 | 0.255 | -5.01 ± 1.80 | 0.007 | -0.02 ± 0.03 | 0.475 |
| Weight loss, % | -0.38 ± 0.31 | 0.215 | -0.56 ± 0.33 | 0.098 | 0.01 ± 0.01 | 0.365 |
| Albumin, *g.L^-1^, median (IQR)* | -0.21 ± 0.4 | 0.590 | 0.67 ± 0.48 | 0.161 | -0.01 ± 0.01 | 0.380 |
| Prealbumin, *g.L^-1^, median (IQR)* | 13.02 ± 23.47 | 0.581 | -22.04 ± 29.45 | 0.457 | -0.08 ± 0.42 | 0.861 |

Health related quality of life is assessed with the EQ-5D-3L (EuroQol Research Foundation https://euroqol.org). Quality of life time trade-off utility values were calculated using the French value set. Perceived health was rated on a visual analog scale (VAS) from 0 (worst) to 100 (best). Dyspnea was assessed by the modified Medical Research Council (mMRC) dyspnea scale.

SAPS, Simplified Acute Physiologic Score; HFNC, high-flow nasal cannula; CPAP, continuous positive airway pressure; NIV, noninvasive ventilation; mMRC, modified Medical Research Council dyspnea scale.

The linear regression coefficients represent the average increase or decrease in the variable to be explained when we compare two subjects with explanatory quantitative variables that differ by one unit or when we compare two subjects with explanatory qualitative variables taking the reference value for one of the subjects and another value for the second subject.

**Table E3. Pulmonary function tests and blood gases at the 2-month assessment: factors associated with reduced exercise capacity and health-related quality of life two months after intensive care unit admission**

|  | **Two-month**  **Six-minute walk test**  **% predicted**  **n=70** | | **Two-month EQ-5D-3L**  **Visual Analog Scale**  **n=69** | | **Two-month EQ-5D-3L**  **Time Trade-Off**  **n=77** |  |
| --- | --- | --- | --- | --- | --- | --- |
|  | Linear regression coefficient ± SD | **P** | Linear regression coefficient ± SD | **P** | Linear regression coefficient ± SD | **P** |
| Total lung capacity^a^*, % of predicted, median (IQR)* | 0.34 ± 0.13 | 0.300 | 0.66 ± 0.14 | 0.637 | -0.003 ± 0.003 | 0.195 |
| Total lung capacity^a^ < 80% of predicted value, *n (%)* | 8.30 ± 4.11 | 0.048 | -1.67 ± 4.70 | 0.723 | -0.03 ± 0.09 | 0.692 |
| Forced vital capacity*, % of predicted, median (IQR)* | 0.36 ± 0.09 | 0.000 | 0.23 ± 0.11 | 0.041 | -0.001 ± 0.002 | 0.185 |
| Forced vital capacity, <80% of predicted, *n (%)* | 0.36 ± 0.09 | <.001 | 0.23 ± 0.11 | 0.041 | -0.003 ± 0.002 | 0.185 |
| Forced expiratory volume in one second*, % of predicted, median (IQR)* | 0.35 ± 0.09 | 0.000 | 0.07 ± 0.12 | 0.565 | -0.009 ± 0.002 | 0.142 |
| FEV_1_/FVC*, %, median (IQR)* | -0.06 ± 0.23 | 0.810 | -0.51 ± 0.22 | 0.023 | -0.011 ± 0.004 | 0.615 |
| FEV_1_/VC <70%*, n (%)* | -13.52 ± 6.40 | 0.038 | 8.50 ± 7.43 | 0.256 | 0.12 ± 0.17 | 0.512 |
| Diffusing capacity for carbon monoxide^b^, *% of predicted, median (IQR)* | 0.54 ± 0.10 | <0.0001 | 0.23 ± 0.14 | 0.114 | -0.001 ± 0.003 | 0.836 |
| Carbon monoxide transfer coefficient^b^, *% of predicted, median (IQR)* | 0.37 ± 0.10 | <0.0001 | 0.22 ± 0.13 | 0.101 | 0.002 ± 0.002 | 0.395 |
| Diffusing capacity for carbon monoxide^b^ < 80% of predicted, *n (%)* | 12.58 ± 4.27 | 0.005 | 3.32 ± 5.07 | 0.515 | 0.04 ± 0.09 | 0.691 |
| Sniff nasal inspiratory pressure^c^, *% of predicted, median (IQR)* | 0.27 ± 0.07 | 0.001 | 0.05 ± 0.08 | 0.568 | 0.002 ± 0.002 | 0.187 |
| Maximal inspiratory pressure^d^, *% of predicted, median (IQR)* | 0.11 ± 0.06 | 0.080 | 0.10 ± 0.07 | 0.140 | 0.001 ± 0.001 | 0.289 |
| Maximal inspiratory pressure <80% of predicted, *n (%)* | 5.81 ± 4.11 | 0.163 | 5.34 ± 4.54 | 0.245 | 0.06 ± 0.09 | 0.531 |
| PaO_2_^e^, *mmHg, median (IQR)* | 0.47 ± 0.17 | 0.007 | -0.08 ± 0.20 | 0.692 | 0.003 ± 0.004 | 0.393 |
| PaCO_2_^e^, *mmHg, median (IQR)* | -0.84 ± 0.58 | 0.156 | -0.06 ± 0.63 | 0.926 | 0.01 ± 0.013 | 0.609 |
| pH^e^*, median (IQR)* | 27.82 ± 79.44 | 0.728 | -122.86 ± 78.40 | 0.123 | -0.53 ± 1.43 | 0.712 |
| SaO_2_^e^, *%, median (IQR)* | 4.13 ± 1.24 | 0.002 | -1.03 ± 1.88 | 0.586 | 0.03 ± 0.03 | 0.383 |

Health related quality of life is assessed with the EQ-5D-3L (EuroQol Research Foundation https://euroqol.org). Quality of life time trade-off utility values were calculated using the French value set. Perceived health was rated on a visual analog scale (VAS) from 0 (worst) to 100 (best). Dyspnea was assessed by the modified Medical Research Council (mMRC) dyspnea scale.

FEV_1_, forced expiratory volume in one second; FVC, forced vital capacity.

^a^Data available for 78 cases, ^b^Data available for 82 cases, ^c^Data available for 76 cases, ^d^Data available for 81 cases, ^e^Data available for 75 cases.

The linear regression coefficients represent the average increase or decrease in the variable to be explained when we compare two subjects with explanatory quantitative variables that differ by one unit or when we compare two subjects with explanatory qualitative variables taking the reference value for one of the subjects and another value for the second subject.

**Table E4. Pulmonary function tests and blood gases at the 2-month and 12-months assessment**

|  | **2-months**  **(n=89)** | **12-months**  **(n=42)** | **P** |
| --- | --- | --- | --- |
|  |  |  |  |
| Six-minute walk test*, m, median (IQR)* | 392 (322 – 484) | 506 (440 – 562) | <0.0001 |
| Normalized six-minute walk test*, %, median (IQR)* | 58 (47 – 69) | 80 (72 – 82) | <0.0001 |
| Total lung capacity*, % of predicted, median (IQR)* | 78 (63 – 89) | 77 (72 – 86) | 0.756 |
| Total lung capacity < 80% of predicted, *n (%)* | 37 (47) | 21 (54) | 0.365 |
| Forced vital capacity*, % of predicted, median (IQR)* | 76 (67 – 93) | 90 (74 – 104) | <0.0001 |
| Forced vital capacity, <80% of predicted, *n (%)* | 39 (45) | 13 (31) | 0.160 |
| Diffusing capacity for carbon monoxide, *% of predicted, median (IQR)* | 56 (45 – 67) | 65 (59 – 75) | <0.0001 |
| Diffusing capacity for carbon monoxide < 80% of predicted, *n (%)* | 54 (69) | 32 (84) | 0.081 |
